# Supplementary material for: Sublethal Concentrations of 2C-I and 25I-NBOMe Designer Drugs Impact Caenorhabditis elegans Development and Reproductive Behavior
Source: Int J Mol Sci. 2025 Mar 26;26(7):3039. doi: 10.3390/ijms26073039 (PMC11988394; doi:10.3390/ijms26073039)
Supplement: Supplementary file 1 [file ijms-26-03039-s001.zip › ijms-3445407-supplementary.pdf]

Supplementary information

Table S1. Concentration of the drugs necessary to reach half the maximum effect - cell death (LC_{50}), Top (maximum effect), Bottom (minimum effect) and Hill slope of the concentration-response curves of 2C-I (0-10 mM) and 25I-NBOMe (0-0.5 mM) in *C. elegans*, 24 hours after exposure. [**** $p < 0.0001$ 2C-I LC_{50} *vs* 25I-NBOMe LC_{50}]. LC_{50} results are presented as mean with 95% Confidence Interval (CI).

	**2C-I**	**25I-NBOMe**
**LC_{50}** half-maximum-effect concentrations, mM 95% CI	**1.374** (1.284 - 1.448)	**0.236** (0.213 - 0.255)
**Top** maximal cell death, % control	96.68	100.0
**Bottom** baseline, % control	9.734	12.20
**Hill slope**	7.360	4.940
**Curve *p* value** comparison between the fitted curves	-	****
